# Early Athletic Identity Formation and Development: Perceptions of Elite Gaelic Athletes

**DOI:** 10.3390/sports13020033

**Published:** 2025-01-24

**Authors:** Marion Geary, Niamh Kitching, Mark Campbell, Frank Houghton

**Affiliations:** 1 Department of Sport and Early Childhood Studies, Technological University of the Shannon: Midlands Midwest, V94 EC5T Limerick, Ireland; 2 Department of Arts Education & Physical Education, Mary Immaculate College, V94 VN26 Limerick, Ireland; niamh.kitching@micl.ul.ie; 3 Department of Physical Education and Sport Sciences, University of Limerick, V94 T9PX Limerick, Ireland; mark.campbell@ul.ie; 4 Department of Applied Social Sciences, Technological University of the Shannon: Midlands Midwest, V94 EC5T Limerick, Ireland; frank.houghton@tus.ie

**Keywords:** identity theory, identity formation, multidimensional identity, athletic identity foreclosure, athlete transitions, GAA

## Abstract

Background/Objectives: Athletic identity (AI) is an important part of an elite athlete’s self-concept and can positively impact sports performance, but over-emphasis can lead to potentially negative outcomes. Identity theory suggests that identity is shaped by individuals’ roles, group affiliations, self-perceptions, and important changes in personal circumstances. Athlete transitions are changes that occur in an athletes’ athletic and/or non-athletic circumstances, which can impact identity development. Using identity theory, this study aimed to explore the athletic transitions important in early AI formation and development in elite Gaelic athletes. Methods: Nine athletes were purposively sampled and participated in semi-structured interviews that were analysed using a six-step thematic analysis. Results: Findings indicate that the initiation, development, and mastery transitions in sport influence AI formation and align with the identity theory precepts of roles, groups, and persons in identity development. AI formation and development is individualised and shaped by perceived athlete competence, associated external validation, and the increased professionalism and personal commitment associated with higher levels of performance. Conclusion: We call for a greater emphasis to be placed on the development of multi-faceted identity profiles in elite Gaelic athletes.

## 1. Introduction

According to the identity theory, an individual’s identity is shaped by the meaning they prescribe to the roles they play (role), the groups they identify with and belong to (group) and their self-perceptions (person) [1,2]. The theory suggests people can hold multiple identities, with each identity’s significance depending on how it aligns with their overall sense of self and social structure [3]. The theory also purports that identity development and change is gradual, and influenced by changes in the person’s situation, conflict between identities causing changes to both, and dissonance between identity meaning and behaviour meaning prompting alignment between the two [1]. Other identity theories also shed light on identity development. Self-determination theory purports that fulfilling basic needs for autonomy and competence nurtures identity resolution, [4] while self-categorisation theory focuses on the self as an individual, group member and human being, with each level forming individual identity elements [5,6]. However, the overarching view of identity theory—that identity is shaped by the reciprocal relationship between social- and self-views—offers the best framework to explore athlete identities and the unique environments in which they exist [7].

Earlier work on identity suggests that role conflict or dissonance may prompt individuals to conform to the expectations of significant others or reference groups resulting in normative orientation or identity foreclosure [8,9]. An individual’s experiences and resultant self-perception, coupled with the desire to protect established identity orientations, can influence role-related behaviours, which may have potentially negative consequences [2,10,11]. Athletes, like others, can develop many salient identities; however, evidence suggests that many develop normative identities based primarily on sport, leading to reduced identity exploration [12]. Athletic identity (AI) is defined as the extent to which an individual bases their self-identity on being an athlete determined by the degree of special attention they devote to being an athlete relative to other identities [13,14]. Empirical evidence about the evolution of AI over time aligns with the foundations of identity theory. Research, for example, suggests the manifestation and development of AI is nuanced based on the importance of sport in the athlete’s life and level of sports performance (i.e., role [15,16,17,18,19]), perceived athlete self-efficacy and how performance impacts self-worth (i.e., person [15,16,20,21,22]), and how identity as an athlete is socially reinforced through cultural and entourage settings, i.e., group [15,16,23]).

The development of AI can be helpful in becoming an elite athlete as it is associated with increased sport self-confidence, motor skill proficiency, successful junior to senior sport transition, and successful sports performance [24,25,26]. Empirical evidence demonstrates that elite athletes can positively engage with multiple identities, and concomitant with identity theory, this engagement is dependent on their individuality, perceived identity salience, conflict resolution between identities, and their social environment [27]. However, because of the many competing roles that elite athletes must navigate, they may find it difficult to develop well-rounded identities, resulting in heightened identification with the athlete role [12]. AI foreclosure occurs when an athlete has strongly committed to the athlete role without any exploration or meaningful engagement with other ideological alternatives [28,29]. Athletes can display strong levels of AI or AI foreclosure, which can be linked to reduced academic performance and career development, burnout, potential loss of self-worth, a loss of a sense of belonging while injured, and challenging transitions within and outside of sport, with some experiencing mental health issues [22,28,29,30].

### 1.1. Athlete Transitions and Identity Development

Transitions in an elite athlete’s career are turning points or changes that can impact their lives within and outside sport, bringing challenges and/or opportunities that they must navigate to cope in sport and other important life roles [25,31,32]. Transitions include moving from junior to senior sport, retirement, injury, and personal and cultural changes. The developmental model of transitions proposes four athletic transitions: initiation (introduction to organised sports, about 6–7 years), development (dedication to sport with increased specialisation, about 12 or 13 years), mastery (intense levels of competition/training, about 18 or 19 years), and discontinuation (retirement, about 28 to 30 years) [33]. These transitions are interconnected with personal, academic, and other athlete life changes, and are more nuanced than these categories suggest, as their timing and nature vary across sports, athletes, and sociocultural contexts [34,35].

The notion in identity theory that a change in a person’s situation (like athlete transitions) can promote identity change is evident in the literature. Findings report the potential benefits of AI in the successful transition from junior to senior sport; however, long term it may be a risk factor for a difficult retirement transition [26,32,36]. Athletes may also experience more immediate issues regarding identity while navigating earlier transitions. For instance, athletes in the development and mastery transitions can experience difficulty in managing academic and athletic responsibilities, with some developing burnout relating to perceived stressors associated with sport and other life demands [37,38]. These difficulties can lead to identity and role conflict, which for some athletes precludes them from developing balanced personal identities at a critical time of identity exploration [39,40].

### 1.2. Elite GAA Athletes and Athletic Identity

Hurling and Gaelic football, traditional Irish sports played by male athletes and governed by the Gaelic Athletic Association (GAA) are part of a wider range of Gaelic games for male and female participants (Gaelic Athletic Association, 2016). ‘Underage’ Gaelic players begin their journey with their local clubs, and those that satisfy certain technical and tactical standards are selected for inter-county academy squads. Most inter-county youth academies will have underage squads (from u13 to u16 years) who play at various non-competitive and competitive levels, varying across the 32 counties of Ireland [41]. Players may then transition to minor (U17s), u20s, and/or senior inter-county level and play in provincial and national-level competitions where All-Ireland finals represent the highlight of the season.

Senior inter-county Gaelic athletes play at the highest level and are considered amateur athletes by the GAA who are bound to the ideal that they represent the pride of their club, community, or county, which is considered sufficient reward for their efforts [42]. Despite this amateur label, inter-county Gaelic athletes identify as elite performance athletes and operate at similar levels to other professional sports based on their utilisation of advances in sport science, strength and conditioning, and coaching [30,43,44]. Recent reports highlighted that 88% of elite Gaelic athletes had four or more training sessions per week, while pitch-based training days accounted for six hours of their day, likened to undertaking a second consecutive shift in work [41,45]. The implementation by the Gaelic games of a new player pathway and sports science framework also reflects a broader cultural shift towards a more professionalised approach [46].

A recent review of Gaelic games psychology literature described identity as a key research area of concern suggesting that elite male Gaelic athletes’ identities are shaped by societal norms, including increasing professionalism [47]. Young Gaelic players navigating the development and mastery transitions in inter-county academies have received attention regarding an over-emphasis on sports performance by management teams leading to athlete disempowerment, burnout, and greater associated AI [30,47]. Furthermore, a mixed methods study examining the AIs of elite Gaelic athletes found that despite the amateur ethos of the GAA, they had high levels of AI and viewed themselves as elite performance athletes [43]. The formation of uni-dimensional identities can be especially challenging for elite Gaelic players as they receive no financial compensation for their athletic efforts, unlike athletes in professional sports [30]. As a result, cultivating academic, vocational, and other multi-dimensional identities is imperative while athletes navigate the various transitions within and beyond sport.

According to identity theory, there is a need to further explore identity formation if we are to better understand identity change, identity adoption, and development through the life course [1]. Significant life transitions can result in identity exploration and evolution, and understanding early AI formation and development can guide practitioners in addressing athletes’ identity aspirations early, reducing later identity challenges [1,12,32,48]. The aim of this study is to examine the athletic transitions important in AI formation and early development in elite Gaelic athletes. Much of the empirical work to date on AI and transitions focuses on changes to athletic identity and pre- and post-retirement [49,50,51,52], the media construction of athlete identities in retirement, [31], and athletic identity in the junior to senior sport transition [25,26,32]. While much of this research explores the impact of these change events on athletic identity, the transition events that influence the early formation and development of AI in elite athletes has not been examined. This study will address this gap in the literature while advancing the existing literature on AI and career transitions.

## 2. Materials and Methods

### 2.1. Philosophical Approach

The pragmatic approach to research emphasises that life experiences are best understood through engagement with human beings with differing perspectives based on context, environment, and emotion [53]. Pragmatism is not concerned with ontology, but suggests that a true reality does not exist and meaningful knowledge or epistemology is founded on the practical uses it has in its given field [54]. This study is underpinned by a desire to inform stakeholders (e.g., parents, athletes, coaches, sports science practitioners), regarding AI formation in elite Gaelic athletes. Pragmatism purports that the research approach chosen should be that which best suits the research question. To this end, participant perspectives regarding their AI identity was supported by the use of qualitative semi-structured interviews because of their capacity to examine issues concerning identity [55].

### 2.2. Participants and Sampling Procedures

Nine elite GAA athletes were purposefully sampled to participate in the study and inclusion criteria were that athletes had to be elite senior inter-county hurling and/or football panellists satisfying the criteria for the definition of competitive elite athletes [56] (see Table 1 for participant profiles). Within-sport (athletes’ highest level of performance in the sport and success and experience at the athletes’ highest level) and between-sport criteria (ranking of sport in the athlete’s country of origin, size of the nation in sporting terms and global competitiveness of the sport) were used to classify the athletes [56]. This sample was used as they would yield information suited to practice-based knowledge (at an elite level) in line with the pragmatic approach to research [57]. Sample size was established by adopting a provisional upper limit (*n* = 10) at the outset of the study under the proviso that a final judgement would be made once the study was more advanced and the decision could be more informed [58,59]. Using the concept of “information power” as a guide (the more relevant information a sample holds, the lower the number of participants required), the research team assessed the study aim and richness of the interview data collected to arrive at a final sample size [59]. This overall approach is in line with Braun and Clarke [60], who suggest that the traditional neo-positivist concept of data saturation is not consistent with the underlying values of reflexive TA. Furthermore, the sample size was also in line with Braun et al. [61] who suggest a minimum of 6 participants for meaningful thematic analysis in sport and exercise research (TA).

### 2.3. Data Collection

Ethical approval was granted for the study from the Technological University of the Shannon: Midwest and consent forms and adult information sheets were sent to and signed by each participant. Strict confidentiality measures were enforced throughout the data transcription and analysis process. These included the secure storage of data in password-protected secure OneDrive locations, the use of pseudonyms, the removal of identifiable features such as locations, dates, coach, and team names from interview transcripts, and restricted access to these transcripts, ensuring only the research team had access throughout the study duration. A preliminary interview guide was developed with questions developed from using the extant literature and the principal investigator experiences in practice. Questions included “At what stage did you begin to see yourself as an athlete?”, “How did the idea of you being an athlete make you feel?”, “Did your realisation that you had ability strengthen your belief in yourself as an athlete? If so, why?”, and “What other elements do you have to your identity?”. Semi-structured interviews enabled the conversation to develop, allowing the athletes the flexibility to express their experiences more deeply and explore any unique emergent issues raised by individual participants.

Interviews were arranged such that they suited the athlete and their busy schedules, and locations enabled uninterrupted engagement. A pilot study guided the principal investigator regarding interview protocol, the suitability of the proposed interview questions and her own interview skills [62]. Interviews were conducted by the principal investigator and took 48 min to 1 h and 20 min (M = 63.9, SD = 10.6) to conduct. Interviews were video and audio recorded, and all interviews were transcribed verbatim for data analysis.

### 2.4. Data Analysis

Data analysis was an iterative, bottom-up, six-step reflexive TA [61]. The principal investigator began the familiarisation process by transcribing interviews verbatim with multiple checks between the recordings and the transcripts (step 1). This was followed by reading and coding of the entire dataset (step 1 and 2). Coding was undertaken using a semantic approach (taking the explicit and surface meaning of the data) to categorise interesting features of the data with sufficient detail to provide interpretation and context [63]. Kitching also coded one of the transcripts and a discussion about interpretation, code similarities, etc., was had with the principal researcher. Sample codes included “athlete perceived that he was one of the better players at 9 to 11 years old” and “athlete viewed himself as an “athlete” at minor grade but not before it”. Step 3 involved the process of identifying potential themes by examining the coded dataset and identifying shared meanings. Codes, for example, that referred to feeling like an athlete because of the commitment levels needed at higher levels of performance had shared meaning and formed the initial theme of “athlete increased personal commitment”. Themes were amended (step 4) through multiple review processes, ensuring they reflected the coded data and the entire dataset while satisfying key criteria such as ensuring they were substantial enough to reflect a standalone theme [61]. Defining and naming themes (step 5) were undertaken ensuring dual criteria (each theme was distinctive enough to tell its own story but form a coherent narrative with other themes consistent with the dataset and research question), and write-up completed the process (step 6) [63]. TA was completed using the NVivo 12 software. The corresponding author used ChatGPT (https://chatgpt.com/) for assistance in refin-ing the final review of the document prior to submission.

### 2.5. Trustworthiness

Personal reflexivity was ensured through disclosure to the participants of the primary investigator’s ‘insider’ status, but also through a written personal reflection of her own identity journey as an inter-county athlete, which allowed for greater personal understanding of the potential to influence the study [64]. Interpersonal reflexivity was considered, particularly the potential for a perceived power dynamic through the principal researcher as academic and athlete participants as students (in different educational institutes or in modules separate to that delivered by the principal investigator) [65]. This was achieved by clarifying the study’s focus and the roles of both researcher and participants at the start of each interview, highlighting the researcher’s insider perspective, and using the pilot study to refine questions for clarity and to avoid misinterpretation.

Interview transcripts were reviewed independently by three of the research team at pilot interview and data analysis stages to elicit discussion regarding information power and sample size, and provide an alternative insight into the data [59,66]. Additionally, the 15-point checklist for TA was used to ensure research rigour through detailed transcription, generation of themes that were coherent, distinctive, and consistent, and interpretation, analysis and write-up of findings in line with the overall philosophical approach [61]. Further reliability was ensured through an independent critical friend who reviewed and challenged the principal researcher through open discussion and dialogue [67].

## 3. Results and Discussion

**Figure 1:**
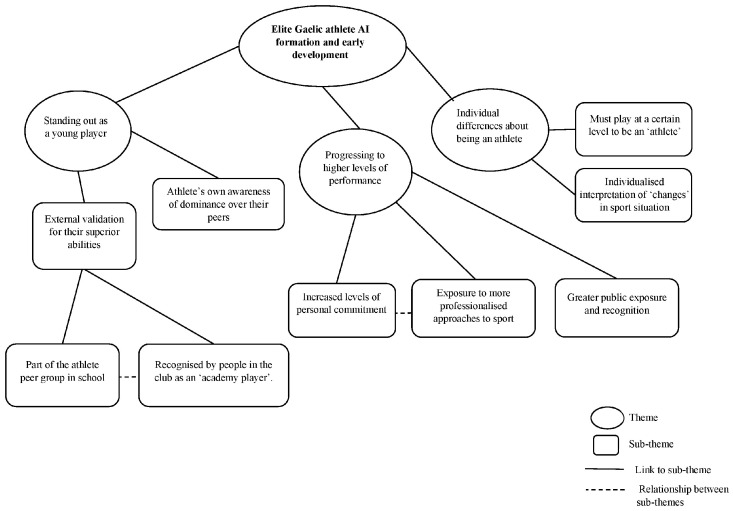
Thematic map showing three key themes.

Several themes were generated regarding athletes’ AI formation and early development. Athletes cited standing out as a young player (leading to participation in underage academies and associated external validation) during the initiation transition and progressing to higher levels of performance (older age groups and adult sport) during the developmental and mastery transitions as key to AI formation. Moreover, athletes varied in their views on what it meant to be an athlete and when it was appropriate to identify as one, which was also important. Figure 1 represents the thematic map arising from the TA process.

### 3.1. Standing out as a Young Player

Several participants believed their AI formation began during the initiation transition (between the ages of 8 and 15) because of their athletic dominance over their peers at club level and the associated external validation they received as a result [33]. One athlete remembered playing at the age of 8 and ‘loving hurling and going down and always scoring goals … and seeing other people weren’t able to rise the ball and stuff like that’ (John). Similarly, another athlete referenced his advanced ability level over his peers at the age of 9 years. He remembered his father (his club coach at the time) trying to keep him grounded but he knew he was one of the better players:

My father…he’d be trying to keep it under wraps…you notice it yourself under 9, 10’s, 11’s that a lot of the game could be revolving around you like, would know yourself if you were a stronger player on the team or not so I suppose around 10, 11 maybe I kinda noticed that I was probably one of the better players on the team…so you knew you were developing then like (Gerard)

Demonstrating proficiency in a different sport was important for another athlete in the study as he was not a recognised Gaelic player in his teenage years. He began to see himself as an athlete at ‘around 15 or 16’ (Robert) because of several trials in professional football clubs in England. He explained that ‘I was playing soccer at quite a high standard like…I went over to England a couple of times… from then…for the soccer part… I thought I was an athlete’.

Exhibiting higher motor skill proficiency than their peers fostered the athletes’ perception of themselves as athletes, which played an important role in AI formation, reflecting previous findings in children and young adolescents [24]. Concomitant with identity theory was that athletes’ ‘role’ as the dominant player as well as the associated self-efficacy (a fundamental aspect of the development of the ‘person’) shaped their identity from a young age [1,2]. Notably, early proficiency in another sport was also important for one participant, suggesting that experiences in one sport can shape identity in another, with athletes already identifying with the athlete role prior to entering a new performance setting [17,68]. Overall, the initiation into sports was an important transition regarding early AI formation in Gaelic games and players who demonstrate early potential may be more inclined towards identifying with the athlete role than their less capable peers [33]. The findings are in line with other studies of high-performance dual careers and retired Olympic athletes who demonstrated AI based on feelings of self-efficacy, with higher levels of AI linked to perceived sports competence and the awareness that they perform at a level that few people can replicate [20,21]. Therefore, if younger athletes transition to higher levels of performance in Gaelic games, it could be suggested that further AI development based on competence and self-efficacy is likely.

Parallel with feelings of self-efficacy was the external validation these young athletes received from peers, parents, and teachers for their superior abilities, with some citing it as important in initial AI formation. Being recognised and known by people in the club as an athlete because of playing on inter-county academy squads was important:

Other parents more than my actual own parents…other people in the club, and as you get older like, you were getting recognised playing like 12, 13, 14 for (the county) development squads so you knew then yourself that you were known as that (an athlete) (Gerard)

The sense of popularity and notoriety amongst peers was also important. Belonging to a ‘group’ was important for one athlete, which was enforced by his perceived status in school. He stated, ‘when you’re seen…when you’re an athlete you have your group of friends…and they’d all be hurlers…it would make you feel good that your kinda, that the athletes would always be the most popular crew” (John). He also recalled moments with his brother when they would play at home, and his brother would “always be like here’s John the hurler”, which further reinforced his self-image as an athlete at an early age.

Social reinforcement, validation, and recognition from significant others and the need for athletes to align their behaviours to match expectations (as per identity theory’s notion of ‘group’) is important in the development and maintenance of AI, and is reflected in findings here [16,21,23]. Social reinforcement and recognition were identified by Stephan and Brewer [21] for example, as important social determinants of maintaining AI in Olympic athletes. Furthermore, athletes participating in sports with higher cultural significance are also characterised by greater overall mean AI scores [17]. Findings here suggest that social recognition and cultural popularity is something that may be important at a much younger age and important in AI formation. The pride and significance of representing one’s local club at intercounty level is deeply ingrained in Gaelic games culture and the social recognition that ensues was an important cultural cue for the adoption of AI despite the young age of some of the participants [30,42,43]. It could be suggested that, culturally, the close-knit nature of the GAA club scene, the perceived privilege associated with playing at county level, and the increasing social media exposure, etc., may only intensify this recognition [17,43]. Care needs to be exercised regarding the perceived notoriety of underage Gaelic academy squads and the intensity of their management. Young players often feel disempowered and obliged to accept demanding training loads, which may negatively impact AI and lead to greater potential for player burnout [30].

Allied to findings in this study, Schmid et al., [49] suggest that the ages of between 10 and 15 years represent a key period for AI formation and development and, thus, participation in academy structures may be meaningful in this regard. Change in a person’s circumstances, as highlighted by the identity theory, can result in identity formation and development, and entry into these academies represents an important change in the young Gaelic athlete’s sporting career [1,30]. Furthermore, the feeling that these young players belonged to salient social groups also served as an important cultural reference point in their AI formation and development. This recognition and feeling of belonging, even at a young age, reinforces identity theory, whereby athletes felt a strong sense of belonging to a ’group’ of talented players [1,2]. The notion that life transitions can prompt important identity work should cue sports professionals and significant others to promote identity awareness and exploration at a young age to encourage adaptive multidimensional identity development [12,69].

### 3.2. Progressing to Higher Levels of Performance

Several athlete’s perceived that early AI development corresponded with their progression to higher levels of performance, e.g., minor or senior intercounty grade, which coincided with the development and mastery transitions [33]. The changes associated with these transitions (and AI formation and development) were increased personal commitment required to perform at these levels, and increased professionalism in team set-ups [1]. In line with the contemporary literature about Gaelic games, the increased professionalisation of the sports was evident in this study at both minor and senior intercounty level [43,47]. Furthermore, the idea in identity theory that changes in a person’s circumstances can result in identity development is evident here [1,3]. Several athletes cited identifying as athletes at 18, corresponding to their inclusion on minor squads and the increased professionalism they experienced. One participant recalled the first time he felt like an athlete:

Didn’t start until maybe around the county minor team, I’d say about 18. And like I know, say I was on a few county underage teams say from 14’s on, but you wouldn’t really call yourself an athlete at that age…you start going to the gym and that, say minor onwards (Paul)

Another participant cited joining the county minor panel at 17 as important; prior to that training was “just” training but at minor level, he was exposed to strength and conditioning and nutrition typical of a more professional approach:

When I was 17 after I made the u16 team and after that I was called up to the minor county panel when I was 17 so I’d say then when we started doing the gym and we started doing actual proper training. We will say u16 you just kinda, you just train and that was it, but this was kinda, you’d diet you know the whole lot’ (Michael)

Other participants cited the professionalised approach at senior intercounty as an important transition in their AI formation as ‘training was way different, was way more professional…it was a huge step up’ (Conor). Another participant did not see himself as an athlete until he experienced the significant increase in the professionalised approach to training at senior inter-county level:

I’d be definitely fitter now than I was when I was 21 say, just good diet and nutrition and all that kind of stuff. And you’re planning to get your sleep in, you’re trying to get your proper foods in, definitely two years ago or three years ago I definitely wouldn’t have done that… I would have been out most weekends… (Ryan)

He also described that “more information was being made available as regards nutrition and how you look after yourself in recovery”, which also furthered his idea of himself as an athlete.

Evidence in other high-performance settings suggests that there is a need to explore the impact of rising through the professional ranks and higher levels of performance on athletes’ AI development, and this study would mirror this assertion [13,18]. Studies in Italian elite and sub-elite student athletes, for example, found more significant levels of AI in elite vs non-elite participants [18]. The marked increase in professionalism at minor and senior inter-county level as perceived by the Gaelic athletes in this study (despite their amateur label) and the parallel association with AI align with the extant literature. Additionally, findings here are in line with identity theory and the notion that changes in personal situation impact identity development [1,3]. The mastery transition can be characterised by athletes with higher AI and improved athletic performance. However, this may also result in a dominant performance narrative, AI foreclosure, and limited identity development, thus, there is a need to support adaptive and multi-dimensional identity development at these junctures [25,28,48].

Increased personal commitment to high-performance sport was also cited as an important factor in AI formation, which for some was evident at minor grade and for others at senior inter-county level. One athlete, for example, acknowledged minor grade as the first conscious identification with the athlete role because of his decision to increase his commitment after a sub-par first year at that level. Being substituted due to an ‘atrocious’ (James) performance signified an important juncture in his AI formation, after which he recalls deciding:

That next year was just going to put in the work, just everything I did, diet, gym, sleep, everything, just was focused that I was going to become a proper athlete and just unbelievable at hurling really, that was my target

Another athlete in the study cited his understanding and acknowledgement of the level of commitment needed to perform at senior inter county level as central in cementing his AI:

That Winter… where we were training 6 nights a week, you know was kind of where I stood back and said you know this is kind of serious enough and this is going to take a lot of commitment and a lot of time which I was quite happy about…it was at that stage I said you know this is where I’m actually an athlete now and where you know it was going to have a massive impact on my life (Dylan)

Exposure to novel and exciting high-performance sports environments (particularly in sports with greater cultural significance like Gaelic games) can promote a singular focus on sport, and with increased sport commitment, performance athletes can be predisposed to higher levels of AI [17]. This may have differing effects. A strong AI is necessary for athletes to make the junior to senior transition (mastery) and it can be a motivational resource for athletes to endure the demands of elite performance [25,32]. Evidence from this study supports the notion that the development of AI may assist athletes navigating the development and mastery transitions, given the importance of increased athlete commitment at minor and senior inter-county levels in AI formation. However, there are also potentially negative implications in relation to greater levels of AI. AI foreclosure, for example, can lead to some athletes questioning their self-worth, a loss of a sense of belonging when injured, burnout, issues managing athletic and life transitions, and long-term implications for athlete mental health and wellbeing in retirement [22,28,29,30]. Based on our findings, one could therefore argue that in line with research, there is also a need to support adaptive identity development at this time [28,48].

### 3.3. Individual Differences About Being an Athlete

The identity theory’s emphasis on the ‘person’ in developing and evolving identities is evident in this study [1,3]. While there were similarities between the athletes regarding AI formation and early development, athletes demonstrated individualised personal beliefs about what it was to be an athlete, which impacted on the transitions that were perceived as important [27]. Two of the participants were talented footballers and had experienced being called for trials for professional clubs in the United Kingdom when they were younger. While one athlete perceived this as central to his AI formation (highlighted previously), for another it had much less perceived significance:

I was supposed to go to England for two weeks…they’d be decent I think they’d be league 1 or something, but, but did I really think I was going to make it as a soccer player? probably not either… (Ryan)

The difference between both athletes’ perceptions of a similar opportunity is reflective of the notion that transitions and identity formation and development are nuanced and individualised [34,35,70]. The differences may be attributed to their experiences as Gaelic players prior to their trials abroad. Ryan, for example, had participated in the underage academy structure in his county in his teenage years, while Robert was not considered a talented underage Gaelic player and, therefore, football may have held more significance for him (see Table 1). Adopting identities based on perceived competence in a domain (i.e., football in the case of Robert), and in activities with heightened perceived importance is well documented and may account for the individual differences seen here [17,33].

Several of the participants had clear opinions in relation to when it was appropriate to consider oneself an athlete, and for two of the athletes it was evident that it was not appropriate to consider themselves as athletes until they had reached the highest level of the sport:

Even though I was on the …minor or U21 teams you know, you don’t really, I wouldn’t really see myself as an athlete at that stage until you get to the top and you’re not at the top until you get to senior level (Dylan)

I was on maybe a few county underage teams say from 14’s on but you wouldn’t really call yourself and athlete at that age…I suppose definitely when you make the county senior team you definitely consider yourself an athlete then (Paul)

The development transition was not considered by these participants as meaningful to be considered an athlete in stark contrast to previous participants who cited the teenage years and minor grade as important. Several potential reasons for these disparities could be suggested. Dylan for example, rose through the academy and senior inter-county structures quickly and was a senior player by the age of 18 (while most of his peers had achieved minor grades only by this stage) (see Table 1). His exposure to higher levels of performance at a younger age may have offered him a different perspective on what it was to be an elite performance athlete [13,18]. Furthermore, both Dylan and Paul were undertaking post-graduate studies, which may have been reflective of the importance they placed on education, and in turn identity development that was less focused on sport [20]. This balanced focus on sport and study may have delayed their association with the athlete identity until later in their sporting careers.

Another athlete alleged that GAA players were ‘totally amateur’ (Ryan) and he believed that he could never consider himself an ‘elite’ performance athlete. One could interpret these findings as indicative of players who espoused the amateur ethos of the Gaelic games and, therefore, may have been reluctant to see themselves as athletes. As noted by Geary et al. [43] this is concerning because athletes may adopt a high-performance lifestyle and associated AI profile without understanding its potential significance in terms of foreclosure or the importance of developing interests outside sport [28,29].

## 4. Conclusions

In conclusion, transitions in sport represent important junctures in the formation and early development of AI in elite Gaelic athletes, reflected by the three key themes generated: standing out as a young player (initiation transition); progressing to higher levels of performance (development and mastery transitions); and individual differences about being an athlete (all transitions). Furthermore, the formation and early development of their AI is supported by the underlying concepts of role, group, and person as espoused by the identity theory [1,2]. AI formation is also individualised and unique to each athlete.

Young Gaelic players who exhibit greater skill levels, athletic dominance, and self-efficacy compared to their peers may begin to develop an athletic identity. This finding highlighted that in conjunction with previous research, AI formation can happen at a young age (potentially during the initiation transition) and is more likely in young athletes who demonstrate greater athletic ability [20,24,33]. The participants’ feelings of being better than their peers also reflected identity theory’s notion of ‘role’ (as one of the better players on the team) and ‘person’ (where increased self-efficacy supported AI development) and ‘group’ (being part of the popular athlete group) [1,2]. Because of their dominance, these players also receive validation from peers, parents, and coaches, etc., which, along with the cultural prestige of inter-county Gaelic games (even at a young age), may foster AI formation [16,21,23,30].

Transitioning to higher levels of performance (development and mastery) was an important progression that fostered early AI development and formation [13]. In line with the cultural shift towards more professionalised approaches to training and coaching in Gaelic games [30,43] athletes cited the increased professionalism associated with minor and senior inter-county set-ups as important in AI formation and development. Further to the increased professionalism of these set-ups was the increased personal commitment needed to perform at these levels, which also encouraged AI formation and development. Identity theory’s view that changes in an individual’s personal situation can foster identity change is evident here and supported by previous research where new and exciting performance environments can result in higher levels of AI [17].

The idea of what it was to be an athlete was individualised and this impacted AI formation and development, which was nuanced depending on the individual [34,35,70]. Personal beliefs about being an athlete influenced which transitions were seen as pivotal to identity development, with some considering senior inter-county level as the minimum standard for being recognised as an athlete [27]. These differences may have been impacted by the broader amateur ethos of the Gaelic games, resulting in a reluctance to identify as an elite performance athlete.

There are limitations to the study that must be recognised. The focus on male-only participants highlights the gender imbalance common in sport and exercise psychology generally, and limits the generalizability of the findings [47,71]. Future research could replicate the study with female athletes or explore gender-based similarities and differences to provide a more comprehensive understanding of AI. Recall bias has been identified as a challenge in research, particularly when participants are asked to recall events from many years prior [17]. In this study, participants recalled key events that occurred over a decade earlier. Experiences stored as memories central to a person’s identity or self-schema are more likely to be recalled with greater accuracy [72,73]. Notably, athletes’ accounts of their AI journeys were recalled without contradictions, despite multiple references during in-depth interviews. Additionally, using recall in qualitative research on athletic identity is not unusual [70,74]. Nonetheless, future research could utilise longitudinal and intervention-based designs to track AI changes over time and identify strategies for positive athlete development throughout their career transitions [12,69].

In line with the pragmatic approach to research, implications for future practice are suggested. Grassroots and academy GAA coaches working with young players could be supported with coach education programmes that incorporate youth psycho-social development. This should include adaptive identity formation, the implications of identity foreclosure, and how to promote multi-dimensional identity development through their practice. Management teams could develop high-performance environments that accommodate and encourage athlete life responsibilities outside sport, e.g., communicating with players about their lives outside sport, avoiding excessive training during busy exam periods, and accommodating work/training conflicts. Sport psychology practitioners working with minor and senior inter-county teams should understand how these high-performance environments affect AI. To support balanced identity development, practitioners should offer both one-on-one and group support, highlighting the benefits and challenges of identifying with the athlete identity and how it might be achieved. Furthermore, parental guidance should be provided in this regard. Finally, the individualised nature of identity development should be acknowledged, and a one size fits all approach should be avoided.

## Figures and Tables

**Table 1:** Participant profiles.

Athlete Pseudonym (*n* = 9)/ Age	Education/Vocation	Athletic Career Trajectory	Current Athletic Status
John (22 years)	2nd year undergraduate student	Part of a traditionally high-performing family in sport Started playing at 4/5 years. Played with a traditional hurling school at secondary level. Played in the academy structure in his county winning an inter-provincial and All-Ireland championship at U21 level. Joined senior inter-county team at 20 years. Won an All-Ireland championship at senior inter-county level	4th year on senior inter-county panel
James (19 years)	2nd year undergraduate student	Part of a traditionally high-performing family in sport Started playing at 3/5 years (firstly with Dad at home and then with the local club). Played in the academy structure (from U14) in his county, winning an inter-provincial championship at minor (U18) grade, and inter-provincial and All-Ireland championship at U21 level. Joined senior inter-county team at 19 years.	1st year on senior inter-county panel
Paul (22 years)	1st year post-graduate student	Part of a traditionally high-performing family in sport Started playing at 4/5 years (firstly with dad at home and then with the local club). Played in the academy structure (from U14) in his county. Joined senior inter-county team at 21 years.	2nd year on senior inter-county panel
Gerard (22 years)	1st year post-graduate student	Started playing at 4/5 years (first in the school yard and then with his local club). Played with a traditional hurling school at secondary level. Played in the academy structure (from age 12 years) in his county. Joined senior inter-county team at 20 years.	2nd year on senior inter-county panel
Dylan (25 years)	2nd year PhD student	Part of a traditionally high-performing family in sport Started playing at 4/5 years (first in the school yard and then with his local club). Played with a traditional hurling school at secondary level. Played in the academy structure (from age 12 years) in his county. Joined senior inter-county team at 18 years. All-Star award recipient (Senior inter-county national team of the year).	7th year on senior inter-county panel
Ryan (21 years)	1st year post-graduate student	Started playing at 5/6 years. Played soccer and trialled in the UK at several professional clubs (15/16 years) Played in the GAA academy structure (teenage years) in his county. Joined senior inter-county team at 18 years.	3rd year on senior inter-county panel
Conor (22 years)	2nd year undergraduate student	Started playing football at 6 years and hurling at 7 years. Played in the academy structure in both hurling and football (from age 12 years) in his county. Joined senior inter-county hurling team at 21 years. Joined senior inter-county football team at 18 years.	Dual Player (1st year on hurling panel/4th year on Gaelic football panel)
Michael (22 years)	4th year undergraduate student	Part of a traditionally high-performing family in sport Started playing at 4/5 years (first with his dad at home and then with his local club). Played in the academy structure (from 16 years) in his county. Joined senior inter-county team at 19 years.	4th year on senior inter-county panel
Robert (22 years)	2nd year undergraduate student	Started playing hurling at 9–10 years (soccer was his main sport growing up) Played soccer and trialled in the UK at several professional clubs (15/16 years) Did not play in the county academy structure—first experience was at U21 level. Joined senior inter-county team at 21 years.	2nd year on senior inter-county panel

## Data Availability

The data generated and analysed for the study are available in the Open Science Framework (OSF) repository at https://osf.io/pv8ud/?view_only=22f6562e45ff49509c5722fc3f3a94e6 (accessed on 10 December 2024).

## References

[1] Stets J.E., Serpe R.T., DeLamater J., Ward A. (2013). Identity Theory. Handbook of Social Psychology.

[2] Stets J.E., Burke P.J. (2000). Identity theory and social identity theory. Soc. Psychol. Q..

[3] Burke P.J., Stets J.E. (2009). Identity Theory.

[4] Faye C., Sharpe D. (2008). Academic motivation in university: The role of basic psychological needs and identity formation. Can. J. Behav. Sci..

[5] Reimer N.K., Schmid K., Hewstone M., Al Ramiah A., D. Chadee (2022). Self-categorization and social identification: Making sense of us and them. Theories in Social Psychology.

[6] Turner J.C., Hogg M.A., Reicher S.D., Wetherell M.S. (1987). Rediscovering the Social Group: A Self-Categorization Theory.

[7] Alfrey K.L., Waters K.M., Condie M., Rebar A.L. (2023). The role of identity in human behavior research: A systematic scoping review. Identity.

[8] Berzonsky M.D. (1992). Identity style and coping strategies. J. Pers..

[9] Marcia J.E. (1966). Development and validation of ego-identity status. J. Pers. Soc. Psychol..

[10] Tušak M., Faganel M., Bednarik J. (2005). Is athletic identity an important motivator?. Int. J. Sport Psychol..

[11] Thoits P.A., Virshup L.K., Ashmore R.D., Jussim L. (1997). Me’s and we’s: Forms and functions of social identities. Self and Identity: Fundamental Issues.

[12] Chun Y., Wendling E., Sagas M. (2023). Identity work in athletes: A systematic review of the literature. Sports.

[13] Edison B.R., Christino M.A., Rizzone K.H. (2021). Athletic identity in youth athletes: A systematic review of the literature. Int. J. Environ. Res. Public Health.

[14] Brewer B.W., Van Raalte J.L., Petitpas A.J., Lavallee D., Wylleman P. (2000). Self-identity issues in sport career transitions. Career Transitions in Sport.

[15] Brewer B.W., Van Raalte J.L., Linder D.E. (1993). Athletic identity: Hercules’ muscles or achilles heel?. Int. J. Sport Psychol..

[16] Brewer B.W., Van Raalte J.L., Cornelius A.E., Pans M. (2022). Third-Generation quantitative assessment of athletic identity: Clarifying the concept. Int. J. Sport Psychol..

[17] Rasquinha A.M., Cardinal B.J. (2017). Association of athletic identity by competitive sport level and cultural popularity. J. Sport Behav..

[18] Lupo C., Mosso C.O., Guidotti F., Cugliari G., Pizzigalli L., Rainoldi A. (2017). The adapted Italian version of the Baller Identity Measurement Scale to evaluate the student-athletes’ identity in relation to gender, age, type of sport, and competition level. PLoS ONE.

[19] Lupo C., Mosso C.O., Guidotti F., Cugliari G., Pizzigalli L., Rainoldi A. (2017). Motivation toward dual career of Italian student-athletes enrolled in different university paths. Sport Sci. Health.

[20] Cartigny E., Fletcher D., Coupland C., Bandelow S. (2020). Typologies of dual career in sport: A cluster analysis of identity and self-efficacy. J. Sports Sci..

[21] Stephan Y., Brewer B.W. (2007). Perceived determinants of identification with the athlete role among elite competitors. J. Appl. Sport Psychol..

[22] Mooney J., Bethell A., Wagstaff C., White R. (2024). The impact of athletic identity, psychological flexibility, and value consistent living on the mental health and well-being of retired elite rugby players. J. Clin. Sport Psychol..

[23] Carless D., Douglas K. (2013). Living, resisting, and playing the part of athlete: Narrative tensions in elite sport. Psychol. Sport Exerc..

[24] Manalo M.J.A., Roncesvalles M.N.C. (2016). Athletic identity mediates the relationship between motor skill proficiency and physical activity level among adolescents. J. Phys. Educ. Res..

[25] Drew K., Morris R., Tod D., Eubank M. (2019). A meta-study of qualitative research on the junior-to-senior transition in sport. Psychol. Sport Exerc..

[26] Franck A., Stambulova N.B., Weibull F. (2016). Profiles of personal characteristics and relevant pathways in the junior-to-senior transition: A longitudinal study of Swedish athletes. Int. J. Sport Psychol..

[27] Lu L.D., Heinze K.L., Soderstrom S. (2018). Playing multiple positions: Student-athlete identity salience and conflict. J. Intercoll. Sport..

[28] Ryba T.V., Ronkainen N.J., Selänne H. (2015). Elite athletic career as a context for life design. J. Vocat. Behav..

[29] Brewer B.W., Petitpas A.J. (2017). Athletic identity foreclosure. Curr. Opin. Psychol..

[30] Hughes L., Hassan D. (2017). Wearing their chains willingly: Athlete burnout and the case of adolescent Gaelic footballers in Ireland. Int. Rev. Sociol. Sport..

[31] Cosh S., Crabb S., Tully P.J. (2015). A champion out of the pool? A discursive exploration of two Australian Olympic swimmers’ transition from elite sport to retirement. Psychol. Sport Exerc..

[32] Franck A., Stambulova N.B. (2019). The junior to senior transition: A narrative analysis of the pathways of two Swedish athletes. Qual. Res. Sport Exerc. Heal..

[33] Wylleman P., Lavallee D., Weiss M.R. (2003). A developmental perspective on transitions faced by athletes. Developmental Sport and Exercise.

[34] Morris R., Heaney C., Kentzer N., Oakley B. (2021). Transitions on the athlete journey: A holistic perspective. Athletic Development: A Psychological Perspective.

[35] Stambulova N.B., Ryba T.V., Henriksen K. (2020). Career development and transitions of athletes: The International Society of Sport Psychology position stand revisited. Int. J. Sport Exerc. Psychol..

[36] Franck A., Stambulova N.B., Ivarsson A. (2024). Swedish athletes’ adjustment patterns in the junior-to-senior transition: Viewing the study from today’s perspective. Ment. Heal. Sport Phys. Act. Sel. Writ. ISSP Acad. Sci..

[37] Cosh S., Tully P.J. (2014). “All I have to do is pass”: A discursive analysis of student athletes’ talk about prioritising sport to the detriment of education to overcome stressors encountered in combining elite sport and tertiary education. Psychol. Sport Exerc..

[38] Lee K., Kang S., Kim I. (2017). Relationships among stress, burnout, athletic identity, and athlete satisfaction in students at Korea’s physical education high schools: Validating differences between pathways according to ego resilience. Psychol. Rep..

[39] Ryba T.V., Stambulova N.B., Selänne H., Aunola K., Nurmi J.E. (2017). “Sport has always been first for me” but “all my free time is spent doing homework”: Dual career styles in late adolescence. Psychol. Sport Exerc..

[40] Arnett J.J. (2007). Emerging adulthood: What is it, and what is it good for?. Child Dev. Perspect..

[41] Kelly E., Banks J., McGuinness S., Watson D. (2018). Playing senior inter-county Gaelic games. Experiences, Realities and Consequences.

[42] Connolly J., Dolan P. (2013). The amplification and de-amplification of amateurism and professionalism in the Gaelic Athletic Association. Int. J. Hist. Sport.

[43] Geary M., Campbell M.J., Kitching N., Houghton F. (2021). “I’m a hurler…basically just a hurler”: A mixed methods study of the athletic identity of elite Irish Gaelic Athletic Association dual career athletes. Int. J. Sport Exerc. Psychol..

[44] Jackman P.C., Lane A., Tod D., Bird M.D. (2023). I realized it was a different kind of culture to other sports”: An exploration of sport psychology service provision and delivery in Gaelic Games. Sport Psychol..

[45] Lane A. (2015). Never enough time. The Experience of Third Level Student County GAA Players.

[46] Lane A., Ryan D., Madigan S., Kirby K., Kearney P., Moyna N., Martin D., O’Reilly E., Kennedy M., Tuohey E. (2023). The Gaelic Games Player Pathway and Sports Science: 2030 Vision.

[47] Jackman P.C., Lane A., Wells N., Kirby K., Bird M.D. (2023). The psychology of Gaelic games: A co-produced scoping review to inform research, policy, and practice. Int. J. Sport Exerc. Psychol..

[48] Ronkainen N.J., Ryba T.V. (2019). Developing narrative identities in youth pre-elite sport: Bridging the present and the future. Qual. Res. Sport Exerc. Health.

[49] Schmid M.J., Hlasová H., Ronkainen N.J., Conzelmann A., Schmid J. (2024). Leaving elite sport, abandoning athletic identity? Development and predictors of athletic identity post-retirement. Ger. J. Exerc. Sport Res..

[50] Lally P.S. (2007). Identity and athletic retirement: A prospective study. Psychol. Sport Exerc..

[51] Wendling E., Sagas M. (2021). Is There a Reformation Into Identity Achievement for Life After Elite Sport? A Journey of Identity Growth Paradox During Liminal Rites and Identity Moratorium. Front. Psychol..

[52] Martin L.A., Fogarty G.J., Albion M.J. (2014). Changes in athletic identity and life satisfaction of elite athletes as a function of retirement status. J. Appl. Sport Psychol..

[53] Kaushik V., Walsh C.A. (2019). Pragmatism as a research paradigm and its implications for social work research. Soc. Sci..

[54] Morgan D.L. (2014). Pragmatism as a paradigm for social research. Qual. Inq..

[55] Guest G., Namey E.E., Mitchell M.L. (2013). Collecting Qualitative Data: A Field Manual for Applied Research.

[56] Swann C., Moran A., Piggott D. (2015). Defining elite athletes: Issues in the study of expert performance in sport psychology. Psychol. Sport Exerc..

[57] Kelly L.M., Cordeiro M. (2020). Three principles of pragmatism for research on organizational processes. Methodol. Innov..

[58] Sim J., Saunders B., Waterfield J., Kingstone T. (2018). Can sample size in qualitative research be determined a priori?. Int. J. Soc. Res. Methodol..

[59] Malterud K., Siersma V.D., Guassora A.D. (2015). Sample size in qualitative interview studies: Guided by information power. Qual. Health Res..

[60] Braun V., Clarke V. (2019). To saturate or not to saturate? Questioning data saturation as a useful concept for thematic analysis and sample-size rationales. Qual. Res. Sport Exerc. Health.

[61] Braun V., Clarke V., Weate P., Smith B., Sparkes A.C. (2016). Using thematic analysis in sport and exercise research. Routledge Handbook of Qualitative Research in Sport and Exercise.

[62] Majid M.A.A., Othman M., Mohamad S.F., Lim S.A.H., Yusof A. (2017). Piloting for interviews in qualitative research: Operationalization and lessons learnt. Int. J. Acad. Res. Bus Soc. Sci..

[63] Byrne D. (2022). A worked example of Braun and Clarke’s approach to reflexive thematic analysis. Qual. Quant..

[64] Law G. (2019). Researching professional footballers: Reflections and lessons Learned. Int. J. Qual. Methods.

[65] Olmos-Vega F.M., Stalmeijer R.E., Varpio L., Kahlke R. (2023). A practical guide to reflexivity in qualitative research: AMEE Guide No. 149. Med. Teach..

[66] Braun V., Clarke V. (2020). One size fits all? What counts as quality practice in (reflexive) thematic analysis?. Qual. Res. Psychol..

[67] Smith B., McGannon K.R. (2018). Developing rigor in qualitative research: Problems and opportunities within sport and exercise psychology. Int. Rev. Sport Exerc. Psychol..

[68] Rea T., Lavallee D. (2015). An examination of athletes’ experiences of the talent transfer process. Talent Dev. Excell..

[69] Champ F.M., Nesti M.S., Ronkainen N.J., Tod D.A., Littlewood M.A. (2020). An exploration of the experiences of elite youth footballers: The impact of organizational culture. J. Appl. Sport Psychol..

[70] Ronkainen N.J., Kavoura A., Ryba T.V. (2016). Narrative and discursive perspectives on athletic identity—Past, present, and future. Psychol. Sport Exerc..

[71] Walton C.C., Gwyther K., Gao C.X., Purcell R., Rice S.M. (2022). Evidence of gender imbalance across samples in sport and exercise psychology. Int. Rev. Sport Exerc. Psychol..

[72] Snelgrove R., Havitz M.E. (2010). Looking back in time: The pitfalls and potential of retrospective methods in leisure studies. Leis. Sci..

[73] Bluck S., Habermas T. (2000). The life story schema. Motiv. Emot..

[74] Kavoura A., Ryba T.V., Chroni S. (2015). Negotiating female judoka identities in Greece: A Foucauldian discourse analysis. Psychol. Sport Exerc..

